# Similarity Measures for Protein Ensembles

**DOI:** 10.1371/journal.pone.0004203

**Published:** 2009-01-15

**Authors:** Kresten Lindorff-Larsen, Jesper Ferkinghoff-Borg

**Affiliations:** 1 Department of Molecular Biology, University of Copenhagen, Copenhagen Ø, Denmark; 2 Ørsted DTU, Technical University of Denmark, Ørsteds Plads, Lyngby, Denmark; Center for Genomic Regulation, Spain

## Abstract

Analyses of similarities and changes in protein conformation can provide important information regarding protein function and evolution. Many scores, including the commonly used root mean square deviation, have therefore been developed to quantify the similarities of different protein conformations. However, instead of examining individual conformations it is in many cases more relevant to analyse ensembles of conformations that have been obtained either through experiments or from methods such as molecular dynamics simulations. We here present three approaches that can be used to compare conformational ensembles in the same way as the root mean square deviation is used to compare individual pairs of structures. The methods are based on the estimation of the probability distributions underlying the ensembles and subsequent comparison of these distributions. We first validate the methods using a synthetic example from molecular dynamics simulations. We then apply the algorithms to revisit the problem of ensemble averaging during structure determination of proteins, and find that an ensemble refinement method is able to recover the correct distribution of conformations better than standard single-molecule refinement.

## Introduction

Protein structures play an important role in molecular biology, and are used for example in protein engineering studies, in drug design and as basis for understanding biological mechanisms at the molecular level. However, not only the average structure but also the dynamics around this structure plays a role in function [1]. These dynamical features of proteins may be highlighted by presenting ensembles of conformations instead of individual representative structures. Such ensembles can either be determined experimentally [2]–[8] or using methods such as molecular dynamics (MD) simulations [1]. In addition to the intrinsic dynamics of protein molecules, additional variability in experimental structures may also arise from uncertainties and statistical noise during experiments and structure determination. In order to reflect this variability, experimental structures should in general be presented as ensembles of conformations [4], [9], [10].

The ability to compare different protein conformations is an important tool in structural biology [11]. For example, the structural changes that occur between the apo- and ligand-bound forms of a protein may provide important clues as to the mechanism of binding. Also, structural similarity between proteins is often used as a starting point for determining and understanding function. Such comparisons typically rely on the calculation of the root mean square deviation (RMSD), or other structural similarity measures, between the atomic coordinates of the different conformations [12].

In a pioneering study, Brüschweiler extended the RMSD measure to ensembles of conformations [13]. In particular, he defined an inter-ensemble RMSD (eRMSD), whose square value is the average mean square deviation between conformations in two ensembles. A very similar measure has recently been used to compare multiple sets of MD trajectories [14] and different methods for protein structure determination [7]. One drawback of the eRMSD is that in general it is non-zero even when the two ensembles are identical, making it difficult to use the eRMSD quantitatively. Also, as the calculations involve the (isotropically distributed) covariance matrices, the eRMSD potentially neglects important contributions from higher order moments of the distributions of conformations. A related measure that has been used to examine convergence of molecular simulations is also based on the covariance matrix [15], [16], and may hence suffer from the same problems.

We here describe three alternative methods to compare ensembles of conformations. The methods are all based on the idea that two ensembles can be compared by estimating the probability densities underlying the ensembles. Using distance measures for probability densities that are based in information theory we can then provide a direct measure of the similarity between protein ensembles.

We first explore the properties of the methods and validate them by examining approximately normally distributed conformations obtained from MD simulations. We then use the methods to revisit the problem of ensemble averaging during protein structure determination from NMR data[17], [18]. In particular, we explore to what extent structure determination methods are able to recover the correct distribution of conformations using experimental data as input. This is an important problem that has so far received very little attention due to the lack of methods for estimating and comparing distributions of conformations. Using synthetic data we find that an ensemble that has been determined by ensemble-refinement is more similar to a reference ensemble than an ensemble determined using standard single-molecule refinement.

## Methods

### Overall strategy for ensemble comparison

Our strategy towards a quantitative comparison of conformational ensembles is first to estimate a probability density for each ensemble and subsequently to compare these densities. We thus view a particular set of conformations as a sample from an underlying distribution and aim to model this distribution based on the sample at hand. By comparing the probability densities we are not only able to test whether the conformations in the two ensembles are similar, but also whether they occur with the same frequency. The latter point is an important criterion in many applications such as for example in the analysis of convergence of MD simulations [19] as well as of methods for protein structure determination [7], [9].

The problem of comparing two ensembles is thus broken down into two steps [20]. We first estimate the densities from the ensembles and secondly we compare the individual densities. The three methods described further below present three complementary methods for estimating the densities, and we here first focus on how to compare the individual probability densities.

Given two ensembles, *A* and *B*, and an estimate of the corresponding density functions, 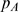 and 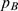, the similarity between *A* and *B* is given as the distance between 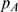 and 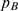. We here use the word distance in a more general meaning including for example measures that may not be a metric in the mathematical sense. Several methods exist for comparing probability densities [21]. Because we want to be able to apply the methods generally we chose measures from information theory that do not rely on a particular form of the probability density. A standard measure of the similarity of two probability densities is the Kullback-Leibler divergence [22], [23]:
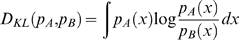
(1)While the Kullback-Leibler divergence is not a true metric it is commonly used as a measure of the similarity between two distributions. In particular 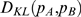 is zero only when 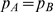, and is positive otherwise.

In an information theory context, 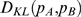 is also known as the relative entropy, and is related to the information lost about 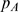 if only 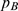 is known [23]. In thermodynamics, the Kullback-Leibler divergence can be thought of as the (non-equilibrium) free-energy difference between the two ensembles [24], [25]. Thus, 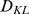 is a natural choice for a method for comparing two protein ensembles. If the score is zero the two ensembles represent the same amount of information and have been derived from the same free energy surface.

A noteworthy property of 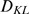 is that it is not symmetric, i.e. in general 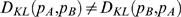. One approach to obtain a symmetric measure is to use the so-called *J*-divergence defined as the average of 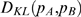 and 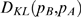 [22], [26].

Another symmetrized, and ‘smoothed’, version of 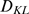 is the related Jensen-Shannon divergence [27], [28]:
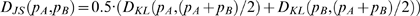
(2)In addition to being symmetric, it can be shown that the Jensen-Shannon divergence is the square of a metric [29]. We in general prefer the Jensen-Shannon divergence because it is well-defined even in the situation where one density is zero in regions where the other is not.

For probability distributions of discrete variables the integrals in the calculations of 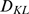 and 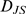 are substituted by summations.

### Calculation of ensemble similarities

We devised three complementary methods for calculating ensemble similarities. These three methods (harmonic ensemble similarity (HES), a clustering based similarity and a dimensionality based similarity) are all described in more detail in the results section and we here provide only some of the more technical details. We applied all three methods to a series of protein ensembles that were generated as described in the subsection *Molecular dynamics and structure determination* below.

First, the harmonic ensemble similarity was calculated using Eq. 7. In those calculations the mean positions of the 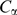 atoms were estimated as the averages over the ensemble. The covariance matrices were estimated using a recently described shrinkage approach [30], [31]. Estimates of the errors of 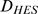 of the full ensembles A–C were obtained as the standard deviation over 100 bootstrap samples [32] from the ensembles. Also, we validated that the values calculated using the shrinkage estimator gave very similar results to the standard calculations of covariances when the ensembles were large.

Secondly, the clustering based ensemble similarities were calculated using the recently described Affinity Propagation (AP) clustering algorithm [33] and a discretized version of Eq. 2 to estimate the Jensen-Shannon divergence between the ensembles. In the case of the three reference ensembles A–C we used the 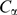 RMSD as input to the algorithm. For the calculations used to analyse ensemble averaging in structure determination we calculated the RMSD over all non-hydrogen atoms. We used the negative RMSD between two conformations as a measure of their similarity. The number of clusters in the AP algorithm is determined by the preference for each conformation to act as a cluster centre, and we varied this number to obtain different number of clusters. The results for the reference ensembles A–C were obtained using preferences between −100 and −0.1, and the results in the study of ensemble refinement were obtained using values between −100 and −0.5. We also tested the clustering method described for analysing convergence [19], and obtained similar results although the method was not as efficient as AP for separating the B and C ensembles.

The final method for calculating ensemble similarities is based on a dimensionality reduction approach and consists of two steps. First, we use the Stochastic Proximity Embedding (SPE) algorithm [34], [35] to represent the high-dimensional conformation space in a low-dimensional subspace. The algorithm attempts to find low dimensional projections that minimize the stress function in Eq. 8 in the results section. By minimizing *S* we obtain low dimensional vectors whose pairwise distances are close to the conformational similarities. The basic step in the SPE algorithm involves an update of the positions of the vectors in the subspace. The size of this update is determined by a learning rate which we decreased from 1.0 to 0.001 over 500 steps. At each value of the learning rate we performed 6·10^6^ update steps for the calculations using ensembles A–C (total of 7500 conformations) and 2·10^6^ update steps for the calculations using the reference and NOE-derived ensembles (total of 2307 conformations). We verified that these values of the parameters in SPE were sufficient to find low values of *S*, and that the results could be reproduced using multiple independent runs.

In the second step of the calculations we use kernel density methods to estimate the densities in the projected subspaces [36]–[38]. The results described here were obtained using Gaussian kernels, and in the case of density estimates in dimensions larger than one we used product kernels. We used a plug-in estimator for the kernel bandwidths [39], but have verified that other kernel types and bandwidth estimates give very similar results.

For comparison with the ensemble RMSD method we calculated the squared value of the eRMSD using its definition [13]:
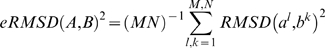
(3)where 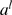 is the *l*'th structure in the *A* ensemble, 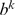 is the *k*'th structure in the *B* ensemble, and *M* (*N*) is the number of conformations in the *A* (*B*) ensemble.

### Molecular dynamics and structure determination

In this section we describe how the different structural ensembles used in the study were generated. For the first validation part of our study we generated three ensembles (A–C) of the GB1 domain of protein G using MD simulations. The starting structure for the simulations was the first model in the PDB entry 3GB1. We used the polar hydrogen model [40] and a potential of mean force description of the solvent [41]. In addition we applied mass weighted harmonic restraints with energy:
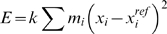
(4)In this equation 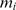 is the mass of the *i*'th atom, 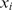 is its position and 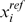 is the position in the reference conformation. The strength of the restraints is determined by *k*, the force constant, chosen to be 0.1, 0.01 and 0.001 in ensemble A, B and C, respectively. The structure was first heated to 300 K over a period of 0.2 ns and then equilibrated for further 2 ns at this temperature before final sampling for 10 ns. Structures were saved every 4 ps giving rise to 2500 structures in each ensemble.

In the second part of our study we analysed to what extent structure determination methods can recover the correct distribution of conformations. We thus generated a reference ensemble of the GB1 domain using an MD simulation with the CHARMM22 topology format and force-field [42] with a modified backbone potential [43] and a generalized Born solvent model [44]. The lengths of bonds involving hydrogen atoms were fixed [45], but no additional restraints were used. The structure was heated to 300 K during 0.2 ns and then equilibrated for 2 ns before sampling for 59 ns. Structures were extracted every 40 ps giving rise to an ensemble consisting of 1475 conformations which we subsequently used to generate synthetic NOE restraints. The native conformation remained stable throughout the simulation. For example, the all-atom (excluding hydrogens) RMSD to the starting conformation is 1.4±0.1 Å (average and standard deviation over the full 59 ns), with no trend of an increasing RMSD during the simulation. In the projections of this ensemble that we describe below, the distribution of conformations appears bimodal. This is caused by a slight structural rearrangement that occurs after 37 ns of the simulation. The rearrangement is localized to residues 10, 11, 40 and 41 which are located close to each other in the structure of Protein G. We note that the corresponding residues in the related GB3 domain have been shown by NMR spectroscopy to display long-timescale motion. The average overall RMSD to the native state is the same before and after the rearrangement.

For the generation of a synthetic NOE dataset we calculated the distances between all pairs of protons in each conformation, and then determined effective distances [46] as 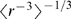. All pairs where this ‘average’ distance was larger than 5 Å were discarded, leaving a total of 2221 distance restraints (symmetry related methyl and ring-protons were treated using pseudo-atoms [47]). Finally, we classified the pseudo-NOE distance restraints as strong (1.8–2.4 Å), medium (2.4–3.8 Å) and weak (3.8–5.0 Å), and these values were then used as lower and upper bounds in structure determination. While this method for estimating NOEs from MD simulations neglects certain dynamical effects [48] it has been shown to work well in practice [49], in particular as the goal is here to generate a synthetic dataset [50] and not to predict experimental NOEs.

The synthetic data were subsequently used as input to a structure determination protocol. To diminish the bias introduced by using the same force field to generate the data and in the structure determination protocol we used a different solvation model [5], [51], [52] in these calculations. Structure calculation was performed using biased MD [53] with NOE distance restraints using a simulated annealing protocol described previously [5]. In these ensemble simulations 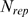 conformations are simulated in parallel. In practice, if 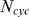 cycles of simulated annealing are carried out one obtains an ensemble consisting of 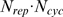 conformations. In this formulation 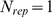 corresponds to standard structure determination methods. We carried out 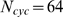, 64, 32, 32 and 16 simulated annealing cycles in simulations with 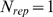, 2, 4, 8 and 16 molecules, respectively, and thereby obtained five different ensembles of GB1.

All simulations were performed using the CHARMM molecular dynamics program [54].

## Results

As described in the Methods section, the overall strategy for comparing structural ensembles is to model each ensemble as a probability distribution, and then to compare these distributions. The previous sections outlined the methods used to compare the probability distributions, and hence the remaining problem is to estimate the densities from the ensembles. The following three sections describe three different, yet complementary, approaches for estimating probability densities from protein ensembles. In each section we describe the underlying idea and apply the method to compare three test-ensembles that we generated to test the methods. In the final part of the Results section we apply all three methods to a common problem relating to ensemble refinement using NMR data.

### Harmonic ensemble similarity

We model the protein ensemble as generated from a probability density function. The form of such density functions are, however, in general unknown and must either be guessed or inferred from the sample. A commonly used approximation is to assume that the ensemble is derived from a multivariate normal distribution [55]. When the parameters in this distribution are estimated from the ensemble, this is known as the quasi-harmonic approximation because anharmonic contributions may be incorporated indirectly via the estimated parameters [55]. In this framework, the probability of observing a conformation, *x*, is given by:
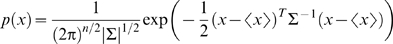
(5)Here *n* is the number of degrees of freedom, 〈*x*〉 are the mean coordinates, Σ is the covariance matrix that contains information about the fluctuations of the ensemble, |Σ| is its determinant and 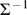 is the inverse of Σ. That is, if we assume that an ensemble *A* is drawn from an underlying Gaussian distribution 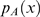, we can estimate 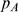 by estimating 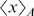 and 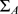 from the ensemble.

When 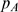 and 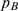 are multivariate normal distributions an analytical solution can be found to the Kullback-Leibler divergence between the two [21], [56].
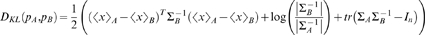
(6)Here 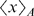 and 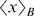 are the means of the two distributions, 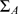 and 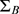 are the covariance matrices, 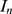 is the identity matrix of size *n*, and *tr*() denotes the trace of a matrix.

We have been unable to derive a closed-form expression for 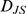 between two normal distributions. Instead we use the symmetrized *J*-divergence defined as the average of 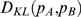 and 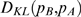. In the case of two multivariate normal distributions we term this average the harmonic ensemble similarity (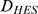):
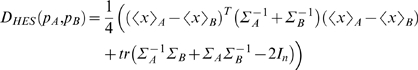
(7)The first term in 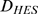 is related to the Mahalanobis distance between the two distributions [57], and is zero only when the two means are identical. This term can be interpreted as a generalized RMSD between the two mean conformations, giving different weights to each atom depending on how much it fluctuates. The second term in 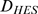 depends only on the covariances of the two ensembles, and is zero when 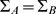. 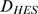 is therefore a measure of the similarity between two ensembles that gives weight to both differences in the mean conformation as well as differences in the fluctuations away from this mean. Thus, 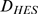 will be zero only when both the mean structures and the fluctuations are identical in the two ensembles. The harmonic ensemble similarity score only explicitly includes the first two moments of the distribution of conformations. In this sense, it is similar in spirit to the eRMSD [13] and the covariance-overlap [16], and may not be applicable for ensembles that have more complicated distributions of conformations. A strength of 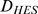 is, however, that it is a direct measure of the similarity of the distribution functions, and therefore has a clear statistical meaning. Further, 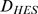 is zero when the two ensembles are identical and non-zero otherwise.

**Figure 1:**
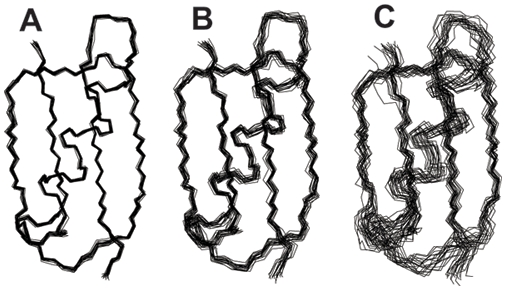
Representative structures from three ensembles generated using molecular dynamics simulations. These ensembles of the GB1 domain of protein G were obtained using MD simulations in the presence of mass-weighted harmonic restraints of increasing size. The three ensembles A, B and C were obtained using force constants 0.1, 0.01 and 0.001, respectively.

In order to demonstrate the ability of 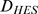 to quantify the similarity between ensembles, we used MD simulations to generate three sets of conformations, termed A, B and C, of the 56 residue GB1 domain of Protein G. In each of the three simulations we applied mass-weighted harmonic restraints to all atoms, and the three simulations differed by the force-constant used for these restraints as follows A (*k* = 0.1), B (*k* = 0.01) and C (*k* = 0.001). As the force constant is decreased, the amplitude of atomic fluctuations become larger and the ensembles thus become increasingly more ‘broad’. We extracted 2500 structures from each MD simulation and all of these structures were used in the calculations described below unless otherwise stated. By construction, the three ensembles were generated so that A and C would both be more similar to ensemble B than to each other. In practice we find that the three ensembles have similar average structures, with the 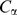 RMSDs of the mean structures being 0.2 Å (A vs. B), 0.7 Å (A vs. C) and 0.6 Å (B vs. C). On the other hand the ensembles differ in how ‘broad’ they are. For example the mean pairwise RMSD within ensemble A is only 0.3 Å, whereas it is 0.5 Å and 0.9 Å for B and C, respectively. Representative structures from the three ensembles are shown in Fig. 1A–C. We note that the harmonic restraints do not ensure that the ensembles conform precisely to a multivariate normal distribution because of the additional presence of the MD force field.

In the comparison of the three ensembles we focus on the 56 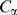-atoms giving a total of *n* = 3·56 = 168 degrees of freedom (*x*,*y*,*z* coordinates for each 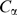-atom). As input to the calculation of 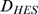 we need to estimate the mean and the covariance matrix of these degrees of freedom from the three ensembles. The mean conformation was estimated as the average over the ensemble. The standard maximum-likelihood method for estimating the covariance matrix, Σ, involves the calculation of each of the matrix elements 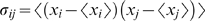 individually. However, while this estimate of Σ is (almost) unbiased for an infinitely large dataset, it is known that a more robust overall estimate for Σ can be obtained using a so called shrinkage estimate when the number of data points is small compared to the number of degrees of freedom [58]. The idea behind the shrinkage approach is that an improved (in terms of total mean square error) estimate can be obtained by simultaneously estimating all elements of the covariance matrix. Importantly, the standard (maximum likelihood) estimate of Σ becomes singular when the number of structures is less than then number of degrees of freedom making it complicated to estimate 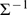 (and |Σ|). As we wish to develop a method that can also be applied to small ensembles such as those determined directly from experiments we therefore used a recently developed shrinkage estimates for the covariance matrices [30], [31], and from these we estimated the similarity between all pairs of ensembles using Eq. 7. These calculations show that ensemble A is more similar to ensemble B (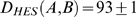) than to ensemble C (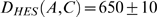), and also that ensemble C is more similar to B (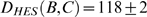) than to A. We also calculated the two terms in Eq. 7 separately and found that they are of comparable magnitude with the first term making up between 40%–58% of 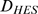. These results are in full agreement with the visual inspection of the ensembles in Fig. 1, and are also in line with the way the ensembles were generated. For comparison, we also calculated the interensemble eRMSD score as described previously [13]: *eRMSD*(*A*,*B*) = 0.45 Å, *eRMSD*(*A*,*C*) = 0.94 Å, and *eRMSD*(*B*,*C*) = 0.91 Å. The eRMSD thus gives a similar ordering as the harmonic ensemble similarity score, although ensemble C is found to be only slightly more similar to B than to A. However, while the self-similarity using 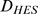 is always zero, this is not the case for the eRMSD: *eRMSD*(*A*,*A*) = 0.26 Å, *eRMSD*(*B*,*B*) = 0.50 Å, and *eRMSD*(*C*,*C*) = 0.92 Å. Taken literally, this means that ensemble *C* is more similar to *B* than to itself, which makes it more complicated to use the eRMSD to rank the different similarities.

In order to demonstrate that the calculations of 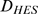 can be carried out using smaller ensembles we repeated the calculations using as little as 25 conformations extracted from each of the full ensembles of 2500 conformations. We denote these subensembles as *A*′, *B*′ and *C*′ and present the results as the average and standard deviation over 100 randomly selected subensembles. The resulting similarity scores are 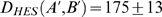, 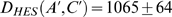 and 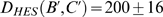. While there are numerical changes from the results of the full ensembles, the overall trends are identical, including the observation that the distance from B to its two ‘neighbouring’ ensembles (A and C) is much smaller than the distance between A and C. These observations are noteworthy as the calculations of 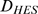 here involve the estimation of the 168×168 covariance matrices from only 25 conformations. In this case the maximum likelihood estimate of Σ would have been singular making it impossible to calculate its inverse and hence 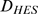. In contrast, the shrinkage estimates of the covariance matrices are guaranteed to be positive definite and hence invertible.

### Ensemble similarity from conformational clustering

Although the calculations of 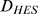 are very fast and straightforward, there may be two potential problems in its practical application. First is the assumption that the ensembles can be described by a multivariate normal distribution. Although anharmonic contributions are partially included by explicitly estimating the covariances from the ensemble, the analytical solution to 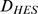 is based on the two ensembles being normally distributed. The second problem in the calculation of 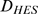 is that in many cases the ensemble size may be considerably smaller than the number of degrees of freedom (*n*). While we have demonstrated above that the *n*×*n* covariance matrix can be estimated from less than *n* conformations using the shrinkage-approach, we have until now only looked at the fluctuations of the 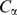 atoms in the ensembles making *n* = 3*N* where *N* is the number of residues. However, in many cases it is of interest to include fluctuations of side chain atoms in the comparison of the ensembles. Even ignoring hydrogen atoms in the GB1 domain, *n*>1000 making the estimation of the covariances from small ensembles difficult.

**Figure 2:**
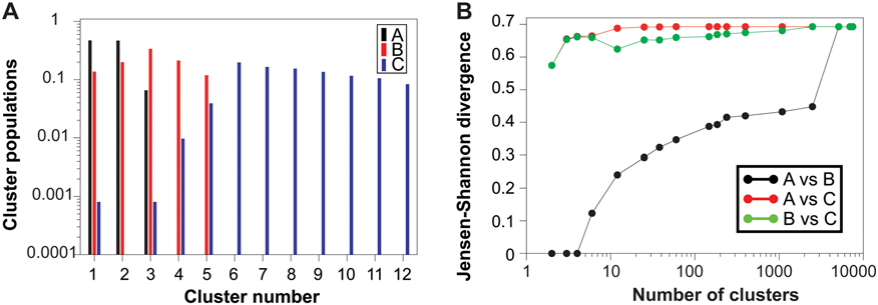
Comparison of the three test ensembles using a method that quantifies the co-occurrence of structures during conformational clustering. A: Populations of each of the ensembles A, B and C in each of the 12 clusters that we obtained using a cluster preference of −10. B: Jensen-Shannon divergence between the three ensembles at a series of cluster preferences giving rise to between 2 and 7500 clusters.

Recently, a method has been proposed to analyse the convergence of MD simulations. The idea is to quantify the co-occurrence of conformations from the first and second half of a simulation when the two halves are clustered together [19]. We here modify and extend this approach to estimate the similarities between two or more ensembles. To illustrate the method we first calculate the 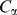 RMSDs between all pairs of the 7500 structures in ensembles A, B and C. We then use a recently described clustering algorithm termed Affinity Propagation (AP) [33] to divide the conformations in to clusters. AP uses the similarities between pairs of structures to decide which conformations should be clustered together. The number of clusters, *k*, is chosen based on the ‘preference’ for each conformation to act as the centre of a cluster. We set the preferences to a common value for all conformations so that all conformations are equally likely to become cluster centres, and vary the preference-value to obtain different levels of clustering. After the structures are clustered we take the population, 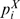, of each ensemble (*X*) in each cluster (*i*) as a probability distribution of conformations. This idea is illustrated in Fig. 2A which shows the populations of ensemble A, B and C in each of the 12 clusters obtained using a common cluster-preference value of −10. At this resolution all structures from ensemble A are found in clusters 1–3, ensemble B populates clusters 1–5, and the broader ensemble C populates 11 of the 12 clusters. Visual inspection of the co-occurrences suggest that the overlap between ensemble A and B is the largest, and further that the overlap between the C and B distributions is larger than between C and A. We then take the cluster populations for an ensemble *X*, 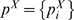, as a density estimate over the discrete set of clusters. From these we then quantify the ensemble similarities by calculating 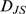 between each pair of ensembles, and find 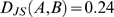, 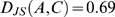 and 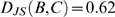 for *k* = 12.

At high values of the common cluster-preference value each conformation ends up in its own cluster and hence *k* = 7500. In this limit the three ensembles do not share any clusters making 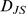 take on its maximal value. At low cluster-preferences all structures group together into very few clusters making 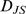 become smaller. In this way, the cluster preference sets the resolution of the algorithm. We therefore repeated the calculations at a range of cluster preferences and calculated 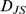 (Fig. 2B). This plot shows that at most resolutions, ensembles A and B are much more similar than the other two pairs when seen from the perspective of co-occurrence in the clusters. On the other hand ensemble C is clearly different from ensembles A and B. At intermediate resolutions it is clear that ensemble C is more similar to ensemble B than to A.

### An ensemble similarity score from dimensionality reduction

One advantage of the clustering method described above compared to the harmonic ensemble similarity score is that it does not require that the ensembles are distributed according to a normal distribution. A potential limitation of the approach is the discreteness of the clusters which means that any conformational differences between structures in the same cluster are ignored. Similarly, any similarities between structures in separate clusters are also ignored. We therefore sought an alternative approach which would provide a continuous description of the distribution of conformations. The main obstacle here is the so called ‘curse of dimensionality’ which highlights the problem of estimating densities in high dimensions without having astronomically large samples [59]. For example, a 168-dimensional histogram with only two bins in each dimension would have more than 10^50^ bins.

Dimensionality reduction methods such as principal components analysis (PCA) and multi-dimensional scaling (MDS) have a long tradition in structural biology, and can be used to represent protein conformations in a subspace of dimension *d* that is much smaller than the full *n*-dimensional configuration space [60], [61]. MDS, for example, takes as input the pairwise similarities, typically an RMSD, between all pairs of conformations and provides a *d*-dimensional projection that aims to preserve these distances as well as possible [60], [62]. Recently, a connection between clustering methods and dimensionality reduction approaches has been found. In particular it has been shown that PCA can be thought of as a continuous solution to the *K*-means clustering problem [63]. This observation suggests that dimensionality reduction may be a natural extension to the clustering method described above, and thereby provide a continuous approach to estimate densities of high-dimensional data from small samples.

An important underlying assumption of methods such as PCA and MDS is that the conformational space is linear. In practical terms this means, for example, that the algorithms give equal weight to an RMSD of 1 Å and 10 Å between two conformations when the projection is constructed, whereas it is clear that large RMSD values are not very useful measures of the similarity between conformations [64]. To overcome this problem one of us has previously applied the non-linear projection method Isomap [65] to represent protein topology space in three dimensions [66]. More recently, Isomap was extended and used to derive order parameters to study protein folding [67].

Other non-linear projection methods exist, and we have here chosen Stochastic Proximity Embedding (SPE) [34] because of its advantageous scaling properties with sample size compared to methods such as Isomap. Because of the stochastic nature of the SPE algorithm it should be run multiple times to test the reproducibility of the results. SPE takes as input the structural similarity between all pairs of conformations, and uses an iterative method to obtain a low-dimensional projection in which pairwise distances are approximately preserved locally. We here define the local neighbourhood as pairs of structures for which the 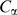 RMSD is less than 1.5 Å, but we have verified that other definitions gave very similar results.

**Figure 3:**
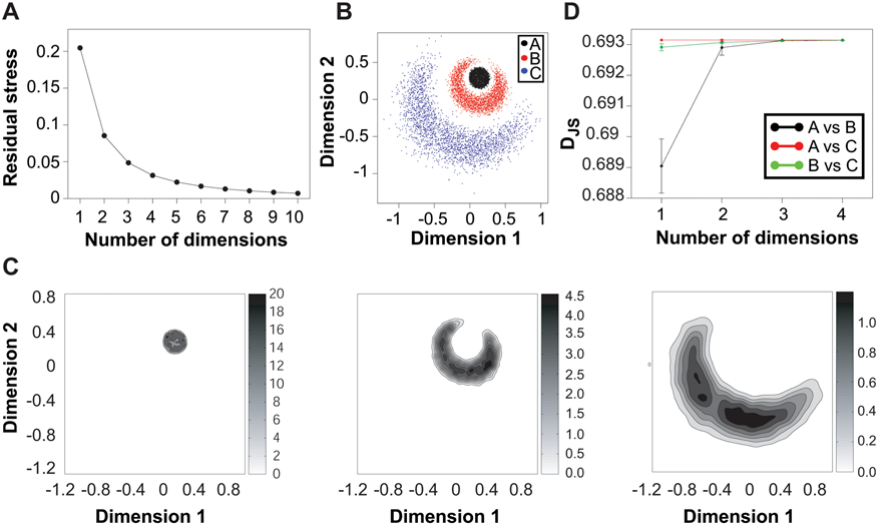
Comparison of the three test ensembles using a method which involves dimensionality reduction and kernel density estimation. A: Average residual stress according to Eq. 8 over 5 independent SPE projections of the three test ensembles. The standard deviation is smaller than the symbols shown. B: Example of a two-dimensional projection of the three ensembles. Each point represents an individual conformation, and the distance between each point is locally approximately the same as the RMSD between those two conformations. The two axes represent the two dimensions in the subspace of the SPE projection. C: Contour plots of the two-dimensional kernel estimates of the densities corresponding to the points in panel B. The grey bars next to the plots indicate the scale of the probability densities. D: Average and standard deviation of the Jensen-Shannon divergence between the three ensembles calculated using the kernel density estimates. The results are shown for different values of the dimensionality of the projections.

The SPE algorithm represents each conformation as a *d*-dimensional vector and finds a collection of vectors so that the Euclidian distance between two vectors in the *d*-dimensional projection is close to the RMSD between those two conformations if they are neighbours of each other (i.e. the RMSD is within a specified cut-off). Only for large values of *d* can all the restraints be fulfilled perfectly. For smaller *d* we calculate the remaining ‘stress’, *S*, as a measure of how well the restraints are fulfilled:
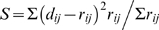
(8)where the sums extend over the neighbours of each conformation [34]. In Eq. 8 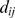 is the Euclidian distance between two points in the low dimensional projection and 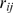 is the RMSD between the two conformations. In Fig. 3A we show the remaining stress as a function of the dimension of the subspace used in the projection. From this figure it is seen that the conformational ensembles can be well represented in a subspace of dimension considerable smaller than *n* = 168. In order to illustrate the low dimensional projections we show in Fig. 3B a scatter-plot of the two-dimensional projection of the 7500 conformations in ensembles A–C. In this plot, each conformation is represented as a single point, and these have been arranged by the SPE algorithm so that structurally similar conformations are located close to each other. As intuitively expected, the conformations in the more ‘narrow’ A-ensemble are located in a small region of the two-dimensional representation of the conformational space. In contrast the broader B- and C-ensembles are spread over larger area of conformational space. It is clear that the SPE algorithm can separate the A, B and C ensembles very efficiently using only the pairwise RMSDs as input. The ‘concentric’ nature of the distributions of conformations is evidence of the fact that the mean structures of the three ensembles are very similar. It is, however, clear that ensembles B and C do in fact not contain conformations that are close to this mean. This is a result of the high dimensional nature of conformational space, which means that the phase space associated with the centre is much smaller than in the outer regions, which therefore contain most of the conformations.

The reduction of the dimensionality of the data makes it feasible to estimate the probability density of each conformational ensemble. Instead of being distributions over the full *n*-dimensional conformation space, they will be distributions over the generalized coordinates in the *d*-dimensional subspace. As we do not want to restrict ourselves to particular distributions of conformations we use non-parametric density estimation to quantify the similarities between the ensembles [20]. In particular, we use kernel density estimation (KDE) [36], [38] to obtain density estimates from the *d*-dimensional samples. In Fig. 3C we show the density estimates obtained for ensembles A–C from the two dimensional projections in Fig. 3B. As also observed in the scatter-plot, the density estimates show that the A-ensemble corresponds to a very sharply peaked probability distribution in line with the stronger harmonic restraints applied during the MD simulation. From the estimated densities we then calculate the Jensen-Shannon divergences between the ensembles. The plot in Fig. 3D shows 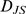, averaged over 5 independent SPE projections, for the comparisons between the A, B and C ensembles as a function of the dimensionality of the projection. As is evident from both the scatter plot and the density estimates there is very little overlap between the three densities, and 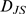 is therefore close to its maximal value. However, the calculations show that ensembles A and B are more similar than the other two pairs of ensembles, and that ensemble C is more similar to B than to A. This is the same ordering of the three ensembles as found in the clustering analysis, although the actual values of 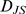 differ in the two calculations.

### Ensemble averaging in structure determination

After validating the methods using the ensembles described above, we then applied the ensemble similarity calculations to an important problem in structural biology. We wanted to examine to what extent it is possible to recover the correct distribution of conformations based only on a set of experimental data, and whether different structure determination approaches differ in their ability to recover the distributions. In particular, we revisit the problem of whether ensemble (multi-conformer) refinement using only NOE distance restraints can be used to recover the conformational distribution of a protein [18]. Again we used the GB1 domain as a test case, and performed a 59 ns reference MD simulation to generate a synthetic pseudo-experimental NOE dataset. The resulting distances were used as restraints in the structure determination of GB1, and the goal is then to examine which of the determined ensembles is most similar to the reference ensemble.

Most standard NOE-based structure determination protocols apply the available distance restraints on to a single conformation. That is, an optimization algorithm (typically simulated annealing) is used to find individual conformations that satisfy as many of the restraints as possible. Through multiple rounds of simulated annealing one then obtains an ensemble of conformations. It is important to note that in this approach it is attempted that all conformations should individually satisfy the restraints. However, these conformations may still differ because of the stochastic nature of the optimization algorithm and because the distance restraints typically do not uniquely identify a single conformation [4], [9], [68].

It is clear, however, that in reality — as well as with the synthetic data generated here — the distance restraints reflect a distribution of conformations, and that no single conformation needs to agree fully with the restraints. In order to reflect this ensemble view of the experimental data, it is possible to use the data in so-called ensemble refinement protocols [2], [3], [17]. In these simulations, the experimental restraints are applied to multiple copies, or replicas, of the protein at any given time and the optimization protocol then aims to find ensembles that satisfy the restraints when the distances are back-calculated as an average over the ensemble. In general, if 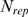 molecules are simulated in parallel none of the individual conformations need to satisfy the restraints individually as long as the 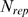 molecules do so as an ensemble.

A practical problem with ensemble simulations is that as 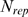 is increased, the number of degrees of freedom in the system also increases because one now attempts to determine 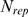 conformations simultaneously. As the total number of distance restraints remains constant, this means that ensemble simulations are more likely to be underdetermined and hence prone to overfitting [7], [17]. It has been suggested that 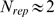 provides an optimal compromise between over- and under-restraining [17]. These conclusions were mainly based on cross-validation as an indirect method for examining whether two ensembles are similar or not, although local similarity scores have also been considered [7]. As 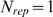 remains the standard method used in protein structure determination we decided to explore this issue further.

**Figure 4:**
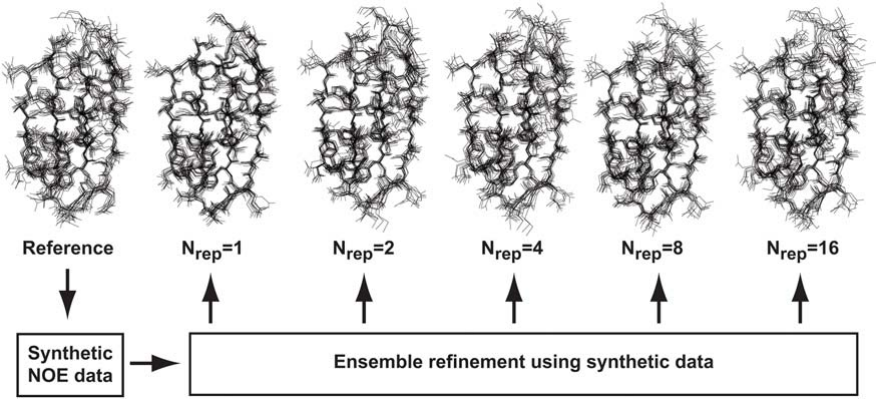
Six ensembles of the GB1 domain of protein G. The reference ensemble was obtained using molecular dynamics simulations, and was used to generate a set of synthetic pseudo-experimental distance restraints. These restraints were subsequently used in either single-conformer refinement (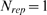) or ensemble refinement using ensemble sizes 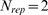, 4, 8 and 16. All non-hydrogen atoms are shown in ten structures from each ensemble.

We used the global ensemble similarity scores described above to quantify whether the ensembles obtained from ensemble simulations (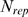 between 2 and 16) are more similar to the ‘true’ reference than those obtained from standard ‘single-conformer refinement’ (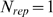). We carried out multiple simulated annealing cycles in simulations with 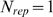, 2, 4, 8 and 16 molecules and thereby obtained five different ensembles of GB1. Ten conformations from each ensemble are shown in Fig. 4. All ensembles are in agreement with the ‘experimental’ NOE restraints as a whole, but only in the 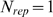 ensemble are all the individual conformations in good agreement with the NOEs. It is noteworthy that the ensembles are visually very similar, and it is not easy to judge which of the five ‘NOE-derived’ ensembles is most similar to the reference MD ensemble.

We first calculated the harmonic ensemble similarity between the reference ensemble and the ensembles determined using 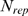 between 1 and 16. Because of the relatively small ensemble sizes we used only the positions of the 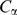 atoms in these calculations. Averaging over 25 randomly selected samples of 64 conformations from each of the NOE-derived ensembles we find that 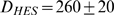, 157±3, 175±5, 212±13 and 233±15 between the MD-reference ensemble and the ensembles determined using 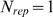, 2, 4, 8 and 16, respectively. These calculations suggest that the ensemble determined using 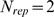 is most similar to the reference ensembles, at least when examining the fluctuations of the 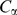 atoms.

To obtain a more detailed view that includes both atoms in the polypeptide backbone chain and in the side chains we proceeded to calculated the pairwise all-atom (excluding hydrogens) RMSD between all 2307 conformations (reference ensemble and five ensembles determined using NOE restraints). These distances were then used as input to the calculations of ensemble similarities using both the clustering and the projection approach.

**Figure 5:**
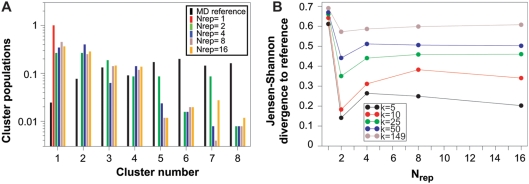
Examination of how well a reference ensemble can be recovered using ensemble simulations. The results shown here were obtained using the clustering method described in the text. A: Populations of each of the ensembles (MD-reference and ensembles obtained using NOE restraints) in each of the 8 clusters found using the affinity propagation clustering algorithm with a cluster preference of −20. B: Jensen-Shannon divergence between the reference ensemble and the ensembles obtained using NOE restraints applied to different ensemble sizes (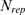). The results are shown for five representative values of the total number of clusters.

We first applied the clustering method to the six ensembles. Figure 5A shows the results of AP clustering at a relatively low value of cluster preferences. In this case the 2307 structures were divided in to eight clusters, and the plot clearly shows that the six ensembles populate the clusters to very different extents. For example, the 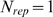 ensemble has all of its structures in cluster 1. In contrast only 2.4% of the reference conformations are found in this cluster.

We repeated the calculations using a range of preference values and calculated 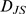 between the reference MD ensemble and each of the five ensembles determined using 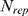 between 1 and 16. In Fig. 5B we show 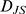 as a function of 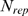 at preference values giving rise to between 5 and 149 clusters. While the numerical values are different at the different levels of resolution, the general trends are very clear. In particular, the ensemble determined using 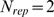 is more similar to the reference ensemble than any of the other ensembles.

**Figure 6:**
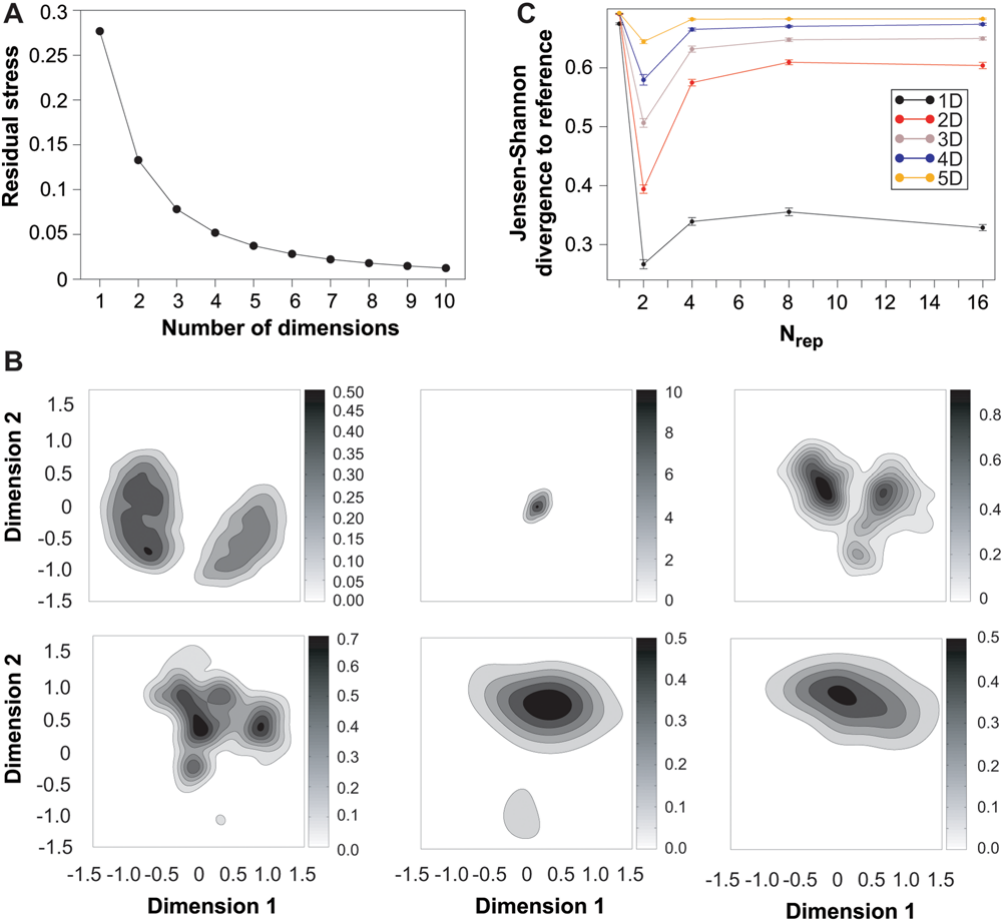
Examination of how well a reference ensemble can be recovered using ensemble simulations. The results shown here were obtained using the projection method described in the text. A: Average residual stress according to Eq. 8 over 10 independent SPE projections of the six ensembles (MD-reference ensemble and five ensembles obtained from NOE restraints). The standard deviation is smaller than the symbols shown. B: Example of the two-dimensional kernel estimates of the densities. The grey bars next to the plots indicate the scale of the probability densities. C: Average and standard deviation of the Jensen-Shannon divergence (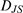) between the reference ensemble and the ensembles obtained using NOE restraints applied to different ensemble sizes (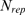). The results are shown for different values of the dimensionality of the projections and are the averages over 10 independent runs of the SPE algorithm.

To analyse further the distributions of conformations in the six ensembles we used SPE to project the 2307 conformations in to low dimensional subspaces. In these calculations we defined neighbouring structures as those that had an all-atom (excluding hydrogens) RMSD less than 1.75 Å, but validated that the conclusions below are the same using other definitions of neighbours (not shown). In Fig. 6A we show the remaining stress, averaged over 10 runs, as a function of the dimension of the projection. As with the harmonic ensembles above, the conformations can be well represented in a low dimensional projection. This is noteworthy because we here use the RMSD between all 436 non-hydrogen atoms corresponding to a much higher dimension of the conformation space. As an example of the distributions we show in Fig. 6B the kernel estimates of the six densities in two dimensions. The first of the plots show the density of the reference ensemble, and its bimodal character is evident. This is caused by a small and localized conformational readjustment for residues 10, 11, 40 and 41 that occurs after 36 ns of the reference simulation. The densities obtained from the conformations determined from the NOE restraints clearly show that the ensembles broaden out as 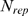 is increased. In particular the ensemble determined using the standard 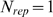 approach is significantly more narrow than the other ensembles. Visual inspection of these densities again suggest that 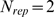 provides an optimal compromise between over- and under-restraining, and is able to recover the bi-modal nature of the conformational ensemble. To quantify these observations, we show in Fig. 6C the Jensen-Shannon divergence between the reference ensemble and the five ensembles for projections in dimensions 1–5. This plot clearly shows that at all dimensions the ensemble determined using 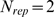 is more similar to the reference ensemble than any of the other four ensembles. Thus, all three ensemble similarity methods show that the ensemble determined using 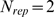 provides the optimal compromise between over- and under-restraining. In contrast, the similarities calculated using the eRMSD method are inconclusive. We find that there is a steady increase in the eRMSD as 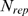 increases, with the similarity between the reference and the ensembles determined using 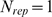,2,4,8,16 being 1.28, 1.36, 1.42, 1.51, and 1.53, respectively. However, as the eRMSD between the reference ensemble and itself is 1.37 it is not clear to us how these numbers should be interpreted.

## Discussion

We have presented three different algorithms to compare two or more ensembles of protein conformations. We first tested the methods using three ensembles, A–C, obtained by MD simulations with additional harmonic restraints. We then proceeded to use the methods to examine how well an ensemble refinement method is able to recover an ensemble of conformations using (synthetic) experimental data.

The first measure we present is the harmonic ensemble similarity. It is based on the assumption of a multivariate normal distribution whose mean and covariance are estimated from the ensembles at hand. Two ensembles are then compared using an analytical expression of the Kullback-Leibler divergence between two normal distributions. The approach is therefore related in spirit to the allosteric potential recently introduced [69], in which the fluctuations are estimated using normal mode analysis. Using this method to compare the three test ensembles we find that both ensembles A and C are significantly more similar to ensemble B than to each other, in full agreement with the way the ensembles were generated. An important advantage of the harmonic ensemble similarity is that it is easy and fast to calculate and has a well defined meaning. Also, because of its analytical nature it is easy to understand the contributions from differences in the mean structure and from changes in fluctuations.

The harmonic ensemble similarity method is based on the assumption of a normally distributed ensemble, and it may not always be clear how to interpret 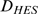 when this assumption is not fulfilled. We note here that the harmonic assumption also forms the basis of methods such as the normal model analysis [55] which is known to describe reasonably well both the amplitude and directionality of native state fluctuations [70]. Also, if only the mean and covariances are known, the multivariate normal distribution is the least biased choice of a density function. Finally, in both of the cases studied here (harmonic ensembles and ensemble refinement), the results obtained using 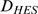 were in full agreement with those obtained using the two other methods described.

A potential limitation in the calculation of 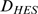 is the uncertainties associated with estimating covariances from sparse data. The shrinkage approach provides a systematic method for estimating the covariance matrix and hence is useful in the common situation when the number of structures is of comparable magnitude to the number of degrees of freedom. In addition, the approach has the added advantage that it produces a regularized and positive definite estimate. Nevertheless, the shrinkage approach can not fully remove the uncertainties associated with estimating covariance matrices from sparse data. Finally we note that the shrinkage method may also be used in others areas of structural biology where estimation of covariance matrices are important. For example, it may be used in the estimation of conformational entropies [55], [71], [72].

In an attempt to evaluate some of the potential practical problems with the harmonic ensemble similarity we devised and tested a similarity score based on clustering the conformations in the ensembles. In particular we used the co-occurrence in conformational clustering as an of estimate for the similarity of two ensembles. In the original implementation of this idea the differences in cluster populations were used as a measure of the similarity of two ensembles [19], but this value may be difficult to interpret [73]. We here substitute this measure by the Jensen-Shannon divergence as a direct measure of the similarity of the two distributions. Also, we use the AP algorithm as a very efficient and fast method for clustering the conformations.

The idea of analysing co-occurrence in clusters was originally designed to examine the convergence of molecular simulations, and hence mainly to answer the question of whether two sub-ensembles are likely to originate from the same distribution [19], [73]. However, our goal is different in that we aim to derive a score that will quantify the divergence between two ensembles. As noted [19], the method is well suited to determine that two ensembles are different, but it is not easy to verify that two ensembles are similar. The results in Fig. 2B show this observation very clearly. At low numbers of clusters the algorithm cannot distinguish ensembles A and B, and hence the distance between these two ensembles is very low despite the fact that they differ considerably in their variability. Nevertheless, one can use the method to quantify the similarity of two ensembles *at the chosen clustering level*. The limitations of the clustering method arise because of the discrete nature of the clusters over which the ensembles are compared, so that conformations in the same cluster are considered equivalent irrespectively of how similar they are.

In order to overcome these potential problems we devised an approach to estimate the density function over a set of continuous variables. In this way we hoped to solve the problems associated with the discreteness of the clusters. Because of the high dimensionality of conformational space it is very difficult to estimate the densities directly [59]. As our main goal is not to estimate the probability densities of the ensembles, but rather to use the density estimates to compare two ensembles, we therefore turned to a dimensionality reduction approach in order to find ‘order parameters’ that would automatically describe the conformations in a subspace of reduced dimensionality. In particular, it has previously been found that the effective dimensionality of conformational space may be significantly smaller than the number of degrees of freedom [60], [61], [74], and we therefore expected that we could capture the conformational distributions well using dimensionality reduction. The results on native state ensembles of the GB1 domain described here show that the ensembles can indeed be well represented in low dimensional subspaces. As with the clustering method there is a compromise between having sufficient resolution, here meaning choosing the dimensionality, and being able to estimate densities. For the test ensembles A–C we find that the method can rank the similarities, and that the results are in accordance with how the ensembles were generated.

Finally we applied the similarity measures to reexamine the problem of ensemble averaging in structure determination. This is an important problem as it is well known that a wrong interpretation of the dynamical contribution to distance restraints may cause errors in structure determination [75]. While ensemble refinement in principle can be used to include dynamical effects, one limitation is that such calculations are more prone to being underrestrained than standard single-molecule refinement is.

An important and general method to compare different structure determination protocols is to generate synthetic data and use this as input to the different protocols. The idea is then to compare the ensembles obtained from structure determination with that used to generate the synthetic data. Until now, such comparison have mainly been carried using validation with independently determined data, cross-validation, measures of the local structural similarity [7], [8], [17], or using reaction coordinates chosen by hand [76], [77]. However, these approaches are only indirect methods for quantifying to what extent one can recover the correct distribution of conformations, and do not take full advantage of the fact that all structural details of the reference ensemble are known.

We here use the ensemble similarity scores as a direct method for quantifying how well the reference ensemble can be recovered. The results show clearly that not taking averaging in to account, i.e. having 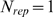, gives rise to an ensemble that is more narrow than the reference ensemble. Increasing values of 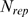 gives broader ensembles, and all three similarity scores show that with the dataset used here, 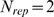 provides the optimal compromise between over- and under-restraining. We emphasize that this result is not necessarily general and applies only to the dataset for GB1 that we used. Also, the dataset that we have generated may be unrealistically large, and different results may therefore be obtained using fewer NOEs and in the presence of systematic errors arising for example from wrong assignments. Finally, the projection of the reference ensemble revealed that the distribution of conformations is bimodal because of a small structural change that occurred during the MD simulation. While this change is very small and localized, it may provide an additional reason for why 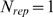 is insufficient to recover the correct distribution. Similarly large conformational fluctuations are, however, likely to occur in real proteins and we note that the residues that display slow motion in our simulation correspond to residues that have been found experimentally to have long-timescale motion in a related protein. Other authors [7], [17] have also suggested 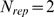 as being optimal for NOE data, at least when there is sufficient experimental data, although larger values may be needed for other data types [7], [78], [79]. Further, we stress that the methods we have presented are completely general and can therefore be used to examine these questions in more detail.

As standard structure determination protocols correspond to 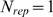, most structures in the Protein Data Bank have been determined in this way. It is therefore of relevance to analyse how well structures determined in this way represent the underlying ensemble. In particular it is relevant to analyse how well such structures represent a ‘typical’ structure from the correct ensemble. The studies described here have not been aimed at examining this question, and may be biased by the bi-modal nature of the reference ensemble used to generate the restraints. However, for this ensemble two lines of evidence point towards the possibility that structure determination using 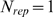 may introduce a bias. First, examining Fig. 5 it is clear that the first cluster contains all of the conformations in the ensemble generated using 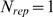, whereas this cluster has the lowest population for the reference cluster. Secondly, the two-dimensional density estimate of the 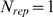 ensemble in Fig. 6 is sharply peaked in a region where there is very little density in the reference ensemble. Together, these observations suggest that, in the case studied here, the 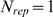 is not only too ‘precise’ (ensemble is too narrow), but may also be somewhat ‘inaccurate’ (centre of distribution does not coincide with that of the reference ensemble). These issues should be examined in more detail, but suggest for example that optimizing force fields against structures from the PDB may introduce biases in the estimated parameters. A related problem is that highlighted by the projections in Fig. 3. While the three ensembles here have similar average structures, only the narrow A-ensemble has structures that are very similar to the average. Thus, even if the average conformation can be determined accurately, it may not be sufficiently representative of the underlying high-dimensional probability distribution to be used in for example structure based drug-design.

The three ensemble comparison methods that we present each have different strengths. The harmonic ensemble similarity is simple to calculate and is based on an analytical relationship (Eq. 7). The equation clearly highlights the fact that for two ensembles to be similar both their ‘average’ structure as well as the fluctuations away from this should be similar. Importantly, the computational complexity in the calculation of the harmonic ensemble similarity is only linear in the number of conformations. In contrast, in the current implementations of the two other similarity scores the algorithms require all pairwise RMSDs, the calculations of which may become prohibitively expensive for very large ensembles. For these reasons we suggest that the harmonic ensemble similarity should be used as a starting point for comparing protein ensembles. In cases where significant deviations from the a normal distribution are expected (or found) we suggest to supplement the calculations of 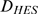 with the clustering and projection based similarity scores. These two methods were designed in different ways to deal with the high-dimensional nature of the data, and hence provide complementary views of the ensemble similarities.

We hope that the methods we present will be of use in many areas of structural biology. For example, the idea of using reference ensembles to validate structure determination protocols has recently been applied to non-native states of proteins [76], [77] as well as revisiting ensemble refinement against Xray diffraction data [8]. Also, until now no community wide standards exist for assessing the convergence of simulations [80]. We suggest that measures such as those presented here could be used to examine the convergence of biomolecular simulations in cases where multiple states may be present. The algorithms can also be used to compare different simulation protocols [81] or molecular force fields [14]. Finally, we hope that the ideas presented here will be used to quantify biologically important changes in conformational distributions that may occur during for example ligand binding [82] and enzyme catalysis [83], and that may form the molecular basis for allosteric effects [84].
